# Redetermination of katayamalite, KLi_3_Ca_7_Ti_2_(SiO_3_)_12_(OH)_2_

**DOI:** 10.1107/S1600536813016620

**Published:** 2013-06-22

**Authors:** Marcelo B. Andrade, Donald Doell, Robert T. Downs, Hexiong Yang

**Affiliations:** a Department of Geosciences, University of Arizona, 1040 E. 4th Street, Tucson, Arizona 85721-0077, USA; b 122 Dublin Street, Peterborough, Ontario, K9H 3A9, Canada

## Abstract

The crystal structure of katayamalite, ideally KLi_3_Ca_7_Ti_2_(SiO_3_)_12_(OH)_2_ (potassium trilithium hepta­calcium dititanium dodeca­silicate di­hydroxide), was previously reported in triclinic symmetry (*C*-1), with isotropic displacement parameters for all atoms and without the H-atom position [Kato & Murakami (1985 ▶). *Mineral. J.* **12**, 206–217]. The present study redetermines the katayamalite structure with monoclinic symmetry (space group *C*2/*c*) based on single-crystal X-ray diffraction data from a sample from the type locality, Iwagi Island, Ehime Prefecture, Japan, with anisotropic displacement parameters for all non-H atoms, and with the H atoms located by difference Fourier analysis. The structure of katayamalite contains a set of six-membered silicate rings inter­connected by sheets of Ca atoms on one side and by an ordered mixture of Li, Ti and K atoms on the other side, forming layers which are stacked normal to (001). From the eight different metal sites, three are located on special positions, *viz*. one K and one Li atom on twofold rotation axes and one Ca atom on an inversion center. The Raman spectrum of kataymalite shows a band at 3678 cm^−1^, similar to that observed for hydroxyl-amphiboles, indicating no or very weak hydrogen bonding.

## Related literature

For previous work on katayamalite, see: Kato & Murakami (1985 ▶). For minerals isostructural with or similar to katayamalite, see: Dusmatov *et al.* (1975 ▶); Fleischer *et al.* (1976 ▶); Menchetti & Sabelli (1979 ▶); Baur & Kassner (1992 ▶); Pautov *et al.* (2010 ▶). For Raman spectroscopic measurements on cyclo­silicates and amphyboles, see: Alvarez & Coy-Yll (1978 ▶); Hawthorne (1983 ▶); Kim *et al.* (1993 ▶); Yang & Evans (1996 ▶); Frost & Pinto (2007 ▶).

## Experimental

### 

#### Crystal data

KLi_3_Ca_7_Ti_2_(SiO_3_)_12_(OH)_2_
*M* *_r_* = 1383.38
Monoclinic, 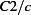

*a* = 16.9093 (10) Å
*b* = 9.7287 (5) Å
*c* = 20.9019 (12) Å
β = 112.396 (3)°
*V* = 3179.1 (3) Å^3^
*Z* = 4
Mo *K*α radiation
μ = 2.36 mm^−1^
*T* = 293 K
0.06 × 0.05 × 0.05 mm

#### Data collection

Bruker X8 APEXII CCD diffractometer
Absorption correction: multi-scan (*SADABS*; Bruker, 2007 ▶) *T*_min_ = 0.871, *T*_max_ = 0.891
26611 measured reflections
4873 independent reflections
3632 reflections with *I* > 2σ(*I*)
*R*_int_ = 0.052

#### Refinement

*R*[*F* ^2^ > 2σ(*F* ^2^)] = 0.035
*wR*(*F* ^2^) = 0.083
*S* = 1.01
4873 reflections
290 parameters
1 restraint
All H-atom parameters refined
Δρ_max_ = 0.83 e Å^−3^
Δρ_min_ = −0.56 e Å^−3^

### 

Data collection: *APEX2* (Bruker, 2007 ▶); cell refinement: *SAINT* (Bruker, 2007 ▶); data reduction: *SAINT*; program(s) used to solve structure: *SHELXS97* (Sheldrick, 2008 ▶); program(s) used to refine structure: *SHELXL97* (Sheldrick, 2008 ▶); molecular graphics: *Xtal*-Draw (Downs & Hall-Wallace, 2003 ▶); software used to prepare material for publication: *publCIF* (Westrip, 2010 ▶).

## Supplementary Material

Crystal structure: contains datablock(s) I, global. DOI: 10.1107/S1600536813016620/wm2749sup1.cif

Structure factors: contains datablock(s) I. DOI: 10.1107/S1600536813016620/wm2749Isup2.hkl

Additional supplementary materials:  crystallographic information; 3D view; checkCIF report

## Figures and Tables

**Fig. 1.**
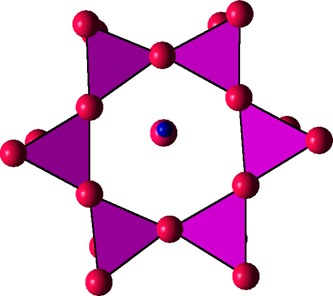
Six-membered silicate ring in katayamalite. Pink, red, and blue represent SiO4 groups, oxygen and hydrogen atoms, respectively.

**Fig. 2.**
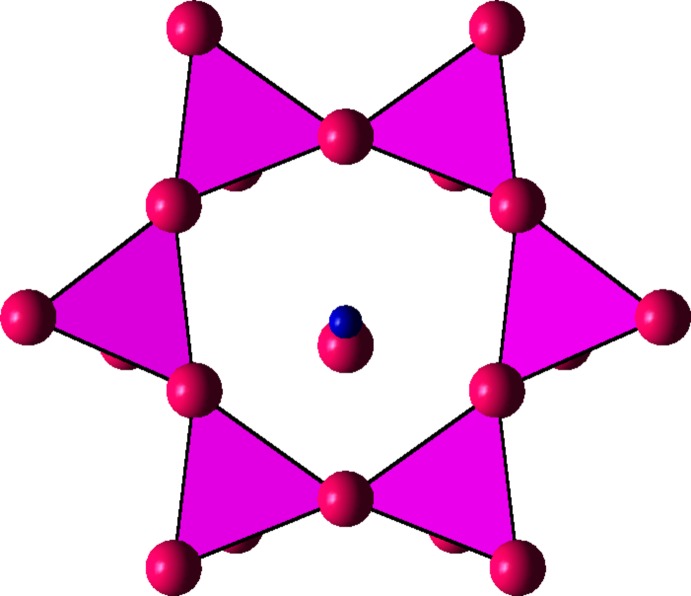
Six-membered silicate ring in tremolite (Yang & Evans, 1996). Pink, red, and blue represent SiO4 groups, oxygen and hydrogen atoms, respectively.

**Fig. 3.**
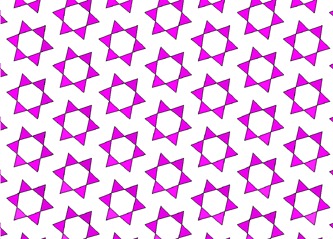
Six-membered silicate rings in the packing of the structure of katayamalite. Pink represents SiO4 groups.

**Fig. 4.**
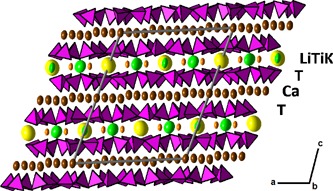
The crystal structure of katayamalite with layers stacked normal to (001). The SiO4 ring layers are connected by Ca atoms on one side and by Li, Ti and K atoms on other side. Pink, brown, green, orange and yellow represent SiO4 groups, Ca, Li, Ti and K atoms, respectively. Displacements ellipsoids are drawn at the 99.999% probability level.

**Fig. 5.**
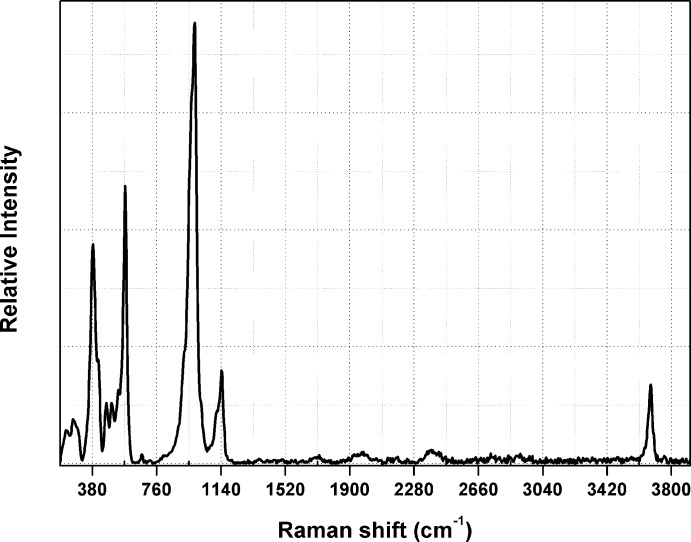
The Raman spectrum of katayamalite.

## supplementary crystallographic information

### Comment

Katayamalite, KLi_3_Ca_7_Ti_2_(SiO_3_)_12_(OH)_2_, belongs to the baratovite group of minerals, which includes katayamalite, aleksandrovite [KLi_3_Ca_7_Sn_2_(SiO_3_)_12_F_2_] (Pautov *et al.*, 2010), and baratovite [KLi_3_Ca_7_Ti_2_(SiO_3_)_12_F_2_] (Dusmatov *et al.*, 1975; Fleischer *et al.*, 1976; Menchetti & Sabelli, 1979).

Kato & Murakami (1985) first described the structure of katayamalite with composition (K_0.89_Na_0.11_)Li_3_Ca_7_(Ti_1.95_Fe_0.05_)(Si_6_O_18_)_2_(OH_1.76_F_0.24_) from Iwagi Island, Inland Sea, Ehime Prefecture, Japan, with triclinic symmetry in space group *C*1 and unit-cell parameters *a* = 9.721 (2) Å, *b* = 16.923 (3) Å, *c* = 19.942 (3) Å, α = 91.43 (10)°, *β* = 104.15 (11)°, γ = 89.94 (10)°. Baur & Kassner (1992), however, proposed that katayamalite is isostructural with baratovite and in fact is monoclinic (space group *C*2/*c*). By using the transformation matrix (0 1 0/ 1 0 0/ -0.5 -0.5 -1) from the triclinic to the monoclinic setting the unit-cell parameters become *a* = 16.923 (3) Å, *b* = 9.721 (2) Å, *c* = 20.909 (3) Å, α = 89.98 (10)°, *β* = 112.40 (10)°, *γ* = 89.94 (10)°. Recently, aleksandrovite, the Sn analogue of baratovite, was described by Pautov *et al.* (2010) in space group *C*2/*c* and with unit-cell parameters *a* = 17.01 (2) Å, *b* = 9.751 (6) Å, *c* = 21.00 (2) Å, *β* = 112.45 (8)°. The structure refinement of aleksandrovite has not been reported yet.

The crystal structure of katayamalite is characterized by layers of close-packed six-membered rings of SiO_4_ tetrahedra (Figs. 1 and 3). There are six non-equivalent Si atoms in the structure. The silicate layers (*T*) are connected by sheets of Ca atoms on one side and by an ordered mixture of Li, Ti and K, on the other side, forming a sandwich (Fig. 4) of *T*–Ca–*T*–(Li,Ti,K) layers. The sandwiches, in turn, are stacked in an *ABAC* packing scheme. The layer of Ca atoms is similar to a brucite layer with its dangling H atoms. The silicate rings are centered by a K atom in one layer, and by an H atom in the other.

The Ca1, Ca2 and Ca3 atoms are eight coordinated and located on general positions while atom Ca4 is 8-coordinated and has 1 symmetry. Li1 lies on a 2-fold rotation axis and Li2 is at a general position; both are tetrahedrally coordinated. The Ti atom is 6-coordinated in form of an octahedron and lies on a general position. The K atom is located on a special position with twofold rotation symmetry; it is 12-coordinated within a distorted coordination environment.

The Si—O bridging bond lengths range from 1.622 (2) to 1.6367 (19) Å, while the Si—O non-bridging bond lengths range from 1.594 (2) to 1.617 (2) Å. The two mean values are 1.629 and 1.604 Å, respectively, while the overall Si—O mean bond lengths is 1.617 Å.

The O—Si—O angles of the six independent SiO_4_ tetrahedra range from 101.60 (10)° to 114.59 (11)°; the smallest angle in each tetrahedron is the one involving two bridging oxygen atoms which are bonded to K. The bridging Si—O—Si angles range from 150.52 (15)° to 158.53 (15)° with a mean value of 154.6°.

The H atom was located by Fourier analysis; it exhibits a short O—H distance (0.68 Å). Also, the (O···O) distances are greater than 3.25 Å revealing no or only little hydrogen bonding interactions.

Fig. 5 displays the Raman spectrum of katayamalite. There have been numerous Raman spectroscopic measurements on a variety of structurally related cyclosilicates. A tentative assignement is made according to previous studies, including tourmalines (Alvarez & Coy-Yll, 1978), benitoite (Kim *et al.*, 1993), and joaquinites (Frost & Pinto, 2007). The bands between 900 and 1000 cm^-1^, and between 1000 and 1200 cm^-1^ are attributable to the Si—O symmetric and anti-symmetric stretching modes, respectively. The strongest band at 570 cm^-1^ is ascribable to the Si—O—Si bending. The bands below 500 cm^-1^ are associated with lattice vibrational modes of Ca—O, Ti—O and K—O. The band at ~3678 cm^-1^ results from the O—H stretching vibrations, indicating little or no hydrogen bonding (Hawthorne, 1983). The band position is quite comparable to that for other hydroxyl-amphyboles. In fact, the O—H configuration in katayamalite and hydroxyl-amphyboles are remarkably analogous (Figs. 1 and 2).

### Experimental

The katayamalite specimen used in this study is from the type locality, Iwagi Island, Inland Sea, Ehime Prefecture, Japan and is in the collection of the RRUFF project (deposition http://rruff.info/R120164). Its chemical composition was measured using a CAMECA SX100 electron microprobe (14 analysis points), yielding the empirical chemical formula, calculated on the basis of 38 anions, (K_0.89_Na_0.12_)_Σ__1.01_Li_3.21_(Ca_6.87_Mn_0.04_Ba_0.02_)_Σ__6.93_(Ti_1.79_Zr_0.14_Fe_0.04_Sn_0.02_)_Σ__1.99_(SiO_3_)_12_(OH_1.55_F_0.45_). The Raman spectrum of katayamalite was collected from a randomly oriented crystal at 100% power on a Thermo Almega microRaman system, using a solid-state 532 nm laser, and a thermoelectrically cooled CCD detector. The laser is partially polarized with 4 cm^-1^ resolution and a spot size of 1 µm.

### Refinement

For simplicity, the ideal chemical formula, KLi_3_Ca_7_Ti_2_(SiO_3_)_12_(OH)_2_, was assumed during the refinement. The structure was refined in space group *C*2/*c*, using the coordinates proposed by Baur & Kassner (1992). The maximum residual electron density in the difference Fourier maps was located at (0.4599, 0.5737, 0.1729), 1.49 Å from K and the minimum at (0.2734, 0.5436, 0.1162) 0.62 Å from Si6. The H-atom was located from a difference Fourier synthesis and its position was refined with isotropic displacement parameters and a soft O—H distance restraint. Reflections (202), (244), (242), (712) and (530) were omitted from the refinement due to large differences between calculated and measured intensities.

### Crystal data

**Table:**

KLi_3_Ca_7_Ti_2_(SiO_3_)_12_(OH)_2_	*F*(000) = 2744
*M**_r_* = 1383.38	*D*_x_ = 2.890 Mg m^−^^3^
Monoclinic, *C*2/*c*	Mo *K*α radiation, λ = 0.71073 Å
Hall symbol: -C 2yc	Cell parameters from 5222 reflections
*a* = 16.9093 (10) Å	θ = 2.7–32.2°
*b* = 9.7287 (5) Å	µ = 2.36 mm^−^^1^
*c* = 20.9019 (12) Å	*T* = 293 K
β = 112.396 (3)°	Block, colourless
*V* = 3179.1 (3) Å^3^	0.06 × 0.05 × 0.05 mm
*Z* = 4	

### Data collection

**Table:**

Bruker X8 APEXII CCD diffractometer	4873 independent reflections
Radiation source: fine-focus sealed tube	3632 reflections with *I* > 2σ(*I*)
Graphite monochromator	*R*_int_ = 0.052
φ and ω scan	θ_max_ = 32.4°, θ_min_ = 2.1°
Absorption correction: multi-scan (*SADABS*; Bruker, 2007)	*h* = −25→25
*T*_min_ = 0.871, *T*_max_ = 0.891	*k* = −14→13
26611 measured reflections	*l* = −24→27

### Refinement

**Table:**

Refinement on *F*^2^	Primary atom site location: structure-invariant direct methods
Least-squares matrix: full	Secondary atom site location: difference Fourier map
*R*[*F*^2^ > 2σ(*F*^2^)] = 0.035	Hydrogen site location: difference Fourier map
*wR*(*F*^2^) = 0.083	All H-atom parameters refined
*S* = 1.01	*w* = 1/[σ^2^(*F*_o_^2^) + (0.0414*P*)^2^ + 0.7329*P*] where *P* = (*F*_o_^2^ + 2*F*_c_^2^)/3
4873 reflections	(Δ/σ)_max_ = 0.001
290 parameters	Δρ_max_ = 0.83 e Å^−^^3^
1 restraint	Δρ_min_ = −0.56 e Å^−^^3^

### Special details

**Table:**

Geometry. All e.s.d.'s (except the e.s.d. in the dihedral angle between two l.s. planes) are estimated using the full covariance matrix. The cell e.s.d.'s are taken into account individually in the estimation of e.s.d.'s in distances, angles and torsion angles; correlations between e.s.d.'s in cell parameters are only used when they are defined by crystal symmetry. An approximate (isotropic) treatment of cell e.s.d.'s is used for estimating e.s.d.'s involving l.s. planes.
Refinement. Refinement of *F*^2^ against ALL reflections. The weighted *R*-factor *wR* and goodness of fit *S* are based on *F*^2^, conventional *R*-factors *R* are based on *F*, with *F* set to zero for negative *F*^2^. The threshold expression of *F*^2^ > σ(*F*^2^) is used only for calculating *R*-factors(gt) *etc*. and is not relevant to the choice of reflections for refinement. *R*-factors based on *F*^2^ are statistically about twice as large as those based on *F*, and *R*- factors based on ALL data will be even larger.

### Fractional atomic coordinates and isotropic or equivalent isotropic displacement parameters (Å^2^)

**Table:**

	*x*	*y*	*z*	*U*_iso_*/*U*_eq_	
K	0.0000	0.07120 (12)	0.2500	0.0361 (3)	
Li1	0.5000	0.0835 (7)	0.2500	0.0167 (15)	
Li2	0.2458 (3)	0.3180 (5)	0.2486 (3)	0.0185 (11)	
Ca1	0.22009 (3)	−0.07260 (5)	0.51311 (3)	0.01101 (12)	
Ca2	0.14530 (3)	0.28347 (5)	0.50710 (3)	0.01108 (13)	
Ca3	0.07248 (3)	0.63859 (5)	0.50005 (3)	0.01062 (12)	
Ca4	0.0000	0.0000	0.5000	0.01050 (17)	
Ti	0.33450 (3)	0.07041 (4)	0.25164 (3)	0.00591 (11)	
Si1	0.61419 (4)	0.26505 (7)	0.36044 (4)	0.00846 (16)	
Si2	0.43082 (4)	0.32346 (7)	0.35974 (4)	0.00856 (16)	
Si3	0.36872 (4)	0.63501 (7)	0.35927 (4)	0.00838 (16)	
Si4	0.49109 (4)	0.88012 (7)	0.36047 (4)	0.00828 (16)	
Si5	0.67395 (4)	0.81556 (7)	0.35909 (4)	0.00837 (16)	
Si6	0.73864 (4)	0.50791 (7)	0.36208 (4)	0.00839 (16)	
O1	0.65854 (12)	0.40272 (18)	0.34511 (11)	0.0148 (4)	
O2	0.65931 (12)	0.22314 (18)	0.44096 (11)	0.0123 (4)	
O3	0.61303 (12)	0.14415 (18)	0.30807 (11)	0.0122 (4)	
O4	0.51486 (12)	0.31007 (19)	0.34026 (11)	0.0139 (4)	
O5	0.35276 (11)	0.23886 (18)	0.30523 (11)	0.0118 (4)	
O6	0.45262 (12)	0.28303 (19)	0.43917 (11)	0.0143 (4)	
O7	0.40985 (12)	0.48821 (18)	0.35090 (12)	0.0163 (5)	
O8	0.27342 (12)	0.65293 (18)	0.30411 (11)	0.0133 (4)	
O9	0.37854 (12)	0.65030 (19)	0.43848 (11)	0.0139 (4)	
O10	0.42704 (12)	0.74837 (18)	0.34065 (11)	0.0139 (4)	
O11	0.52072 (12)	0.92080 (18)	0.44086 (11)	0.0124 (4)	
O12	0.44828 (11)	0.00452 (18)	0.30824 (11)	0.0117 (4)	
O13	0.57262 (11)	0.82069 (19)	0.34535 (11)	0.0151 (4)	
O14	0.72871 (12)	0.85392 (18)	0.43847 (11)	0.0136 (4)	
O15	0.69498 (12)	0.90701 (18)	0.30395 (11)	0.0120 (4)	
O16	0.69232 (13)	0.65696 (18)	0.34465 (11)	0.0153 (4)	
O17	0.78572 (12)	0.47659 (18)	0.30994 (11)	0.0117 (4)	
O18	0.80132 (12)	0.49800 (19)	0.44276 (11)	0.0127 (4)	
O19	0.10128 (13)	0.0692 (2)	0.45750 (11)	0.0132 (4)	
H1	0.097 (2)	0.074 (4)	0.4238 (11)	0.016*	

### Atomic displacement parameters (Å^2^)

**Table:**

	*U*^11^	*U*^22^	*U*^33^	*U*^12^	*U*^13^	*U*^23^
K	0.0352 (6)	0.0342 (6)	0.0410 (8)	0.000	0.0170 (6)	0.000
Li1	0.006 (3)	0.027 (4)	0.016 (4)	0.000	0.004 (3)	0.000
Li2	0.016 (2)	0.015 (2)	0.023 (3)	0.0026 (18)	0.006 (2)	−0.002 (2)
Ca1	0.0084 (2)	0.0101 (2)	0.0156 (4)	0.00000 (18)	0.0057 (2)	−0.0002 (2)
Ca2	0.0081 (2)	0.0104 (2)	0.0155 (4)	0.00035 (17)	0.0053 (2)	0.0002 (2)
Ca3	0.0079 (2)	0.0107 (2)	0.0142 (4)	−0.00007 (17)	0.0054 (2)	0.0002 (2)
Ca4	0.0072 (3)	0.0101 (3)	0.0142 (5)	0.0000 (2)	0.0042 (3)	−0.0008 (3)
Ti	0.00370 (18)	0.00629 (18)	0.0081 (3)	0.00022 (15)	0.00263 (19)	0.00032 (17)
Si1	0.0058 (3)	0.0092 (3)	0.0110 (5)	−0.0005 (2)	0.0040 (3)	−0.0009 (3)
Si2	0.0056 (3)	0.0091 (3)	0.0107 (5)	0.0001 (2)	0.0029 (3)	−0.0008 (3)
Si3	0.0052 (3)	0.0086 (3)	0.0108 (5)	−0.0002 (2)	0.0026 (3)	0.0000 (3)
Si4	0.0049 (3)	0.0096 (3)	0.0101 (5)	−0.0001 (2)	0.0027 (3)	0.0009 (3)
Si5	0.0065 (3)	0.0085 (3)	0.0112 (5)	0.0005 (2)	0.0046 (3)	0.0006 (3)
Si6	0.0068 (3)	0.0084 (3)	0.0112 (5)	−0.0003 (2)	0.0047 (3)	−0.0002 (3)
O1	0.0124 (9)	0.0130 (9)	0.0202 (13)	−0.0047 (7)	0.0076 (9)	0.0007 (8)
O2	0.0107 (9)	0.0143 (9)	0.0123 (12)	0.0003 (7)	0.0052 (9)	−0.0006 (8)
O3	0.0096 (8)	0.0130 (8)	0.0155 (12)	−0.0004 (7)	0.0065 (9)	−0.0036 (8)
O4	0.0069 (8)	0.0185 (9)	0.0176 (12)	0.0024 (7)	0.0060 (9)	0.0004 (8)
O5	0.0076 (8)	0.0130 (8)	0.0129 (12)	−0.0005 (7)	0.0022 (8)	−0.0018 (8)
O6	0.0125 (9)	0.0163 (9)	0.0144 (13)	−0.0010 (7)	0.0052 (9)	−0.0003 (8)
O7	0.0134 (9)	0.0088 (8)	0.0274 (14)	0.0025 (7)	0.0085 (10)	−0.0007 (8)
O8	0.0063 (8)	0.0139 (9)	0.0174 (13)	−0.0013 (7)	0.0021 (9)	0.0010 (8)
O9	0.0119 (9)	0.0163 (9)	0.0132 (13)	−0.0013 (7)	0.0045 (9)	0.0000 (8)
O10	0.0099 (9)	0.0154 (9)	0.0177 (12)	−0.0037 (7)	0.0067 (9)	0.0017 (8)
O11	0.0112 (9)	0.0134 (8)	0.0130 (12)	−0.0006 (7)	0.0050 (9)	−0.0009 (8)
O12	0.0068 (8)	0.0133 (9)	0.0149 (12)	0.0006 (7)	0.0042 (8)	0.0023 (8)
O13	0.0068 (8)	0.0172 (9)	0.0233 (13)	0.0018 (7)	0.0081 (9)	0.0004 (8)
O14	0.0128 (9)	0.0143 (9)	0.0133 (12)	0.0000 (7)	0.0045 (9)	−0.0004 (8)
O15	0.0128 (9)	0.0109 (8)	0.0144 (12)	0.0019 (7)	0.0074 (9)	0.0030 (7)
O16	0.0183 (10)	0.0103 (9)	0.0207 (13)	0.0038 (7)	0.0110 (10)	0.0005 (8)
O17	0.0124 (9)	0.0121 (8)	0.0146 (12)	0.0006 (7)	0.0092 (9)	0.0000 (7)
O18	0.0114 (9)	0.0155 (9)	0.0107 (12)	0.0008 (7)	0.0038 (9)	−0.0001 (8)
O19	0.0143 (9)	0.0152 (9)	0.0140 (15)	−0.0001 (7)	0.0059 (10)	−0.0008 (9)

### Geometric parameters (Å, º)

**Table:**

K—O13^i^	3.083 (2)	Si1—O4	1.6281 (19)
K—O13^ii^	3.083 (2)	Si2—O5	1.604 (2)
K—O4^ii^	3.117 (2)	Si2—O6	1.607 (2)
K—O4^i^	3.117 (2)	Si2—O4	1.626 (2)
K—O1^ii^	3.125 (2)	Si2—O7	1.6367 (19)
K—O1^i^	3.125 (2)	Si3—O8	1.594 (2)
K—O10^ii^	3.141 (2)	Si3—O9	1.607 (2)
K—O10^i^	3.141 (2)	Si3—O10	1.622 (2)
K—O7^ii^	3.142 (2)	Si3—O7	1.6269 (19)
K—O7^i^	3.142 (2)	Si4—O12^xi^	1.606 (2)
K—O16^i^	3.207 (2)	Si4—O11	1.609 (2)
K—O16^ii^	3.207 (2)	Si4—O10	1.6266 (19)
Li1—O12^iii^	1.910 (3)	Si4—O13	1.633 (2)
Li1—O12	1.910 (3)	Si5—O15	1.600 (2)
Li1—O3	1.925 (3)	Si5—O14	1.606 (2)
Li1—O3^iii^	1.925 (3)	Si5—O16	1.6248 (19)
Li2—O15^i^	1.893 (6)	Si5—O13	1.6265 (19)
Li2—O8^ii^	1.904 (5)	Si6—O17	1.605 (2)
Li2—O5	1.906 (5)	Si6—O18	1.617 (2)
Li2—O17^iii^	1.915 (5)	Si6—O16	1.6222 (19)
Ca1—O19	2.348 (2)	Si6—O1	1.6253 (19)
Ca1—O14^iv^	2.3732 (19)	O1—K^xii^	3.125 (2)
Ca1—O9^v^	2.380 (2)	O2—Ca1^vi^	2.3943 (19)
Ca1—O2^vi^	2.3943 (19)	O2—Ca3^xiii^	2.396 (2)
Ca1—O18^i^	2.462 (2)	O2—Ca1^xii^	2.470 (2)
Ca1—O2^i^	2.469 (2)	O3—Ti^iii^	1.924 (2)
Ca2—O19	2.321 (2)	O4—K^xii^	3.117 (2)
Ca2—O18^iv^	2.3901 (19)	O6—Ca3^xiii^	2.395 (2)
Ca2—O14^iv^	2.403 (2)	O6—Ca2^v^	2.416 (2)
Ca2—O6^v^	2.416 (2)	O6—Ca4^xii^	2.4367 (19)
Ca2—O11^i^	2.432 (2)	O7—Ca4^xii^	2.903 (2)
Ca2—O14^i^	2.462 (2)	O7—K^xii^	3.142 (2)
Ca3—O6^vii^	2.395 (2)	O8—Li2^xiv^	1.904 (5)
Ca3—O2^vii^	2.396 (2)	O8—Ti^xiv^	1.9266 (19)
Ca3—O9^viii^	2.397 (2)	O9—Ca1^v^	2.380 (2)
Ca3—O18^iv^	2.4094 (19)	O9—Ca3^viii^	2.397 (2)
Ca3—O11^viii^	2.414 (2)	O9—Ca4^xii^	2.4490 (19)
Ca3—O11^i^	2.4414 (19)	O10—K^xii^	3.141 (2)
Ca4—O19^ix^	2.310 (2)	O11—Ca3^viii^	2.414 (2)
Ca4—O19	2.310 (2)	O11—Ca2^xii^	2.432 (2)
Ca4—O6^i^	2.4367 (19)	O11—Ca3^xii^	2.4414 (19)
Ca4—O6^v^	2.4367 (19)	O12—Si4^xv^	1.606 (2)
Ca4—O9^i^	2.4490 (19)	O13—K^xii^	3.083 (2)
Ca4—O9^v^	2.4490 (19)	O14—Ca1^iv^	2.3732 (19)
Ca4—O7^i^	2.903 (2)	O14—Ca2^iv^	2.403 (2)
Ca4—O7^v^	2.903 (2)	O14—Ca2^xii^	2.462 (2)
Ti—O15^x^	1.9195 (19)	O15—Li2^xii^	1.893 (6)
Ti—O3^iii^	1.924 (2)	O15—Ti^xvi^	1.9195 (19)
Ti—O8^ii^	1.9266 (19)	O16—K^xii^	3.207 (2)
Ti—O17^i^	1.9402 (19)	O17—Li2^iii^	1.915 (5)
Ti—O5	1.9422 (19)	O17—Ti^xii^	1.9402 (19)
Ti—O12	1.9436 (18)	O18—Ca2^iv^	2.3901 (19)
Si1—O3	1.602 (2)	O18—Ca3^iv^	2.4094 (19)
Si1—O2	1.613 (2)	O18—Ca1^xii^	2.462 (2)
Si1—O1	1.6255 (19)	O19—H1	0.680 (18)
			
O12^iii^—Li1—O12	132.6 (4)	O1—Si1—O4	103.60 (10)
O12^iii^—Li1—O3	86.53 (8)	O5—Si2—O6	114.28 (11)
O12—Li1—O3	107.89 (9)	O5—Si2—O4	110.02 (11)
O12^iii^—Li1—O3^iii^	107.89 (9)	O6—Si2—O4	111.16 (11)
O12—Li1—O3^iii^	86.53 (8)	O5—Si2—O7	109.76 (10)
O3—Li1—O3^iii^	144.3 (4)	O6—Si2—O7	108.12 (11)
O15^i^—Li2—O8^ii^	136.3 (3)	O4—Si2—O7	102.86 (11)
O15^i^—Li2—O5	110.5 (3)	O8—Si3—O9	114.47 (11)
O8^ii^—Li2—O5	86.1 (2)	O8—Si3—O10	106.95 (11)
O15^i^—Li2—O17^iii^	86.6 (2)	O9—Si3—O10	110.52 (11)
O8^ii^—Li2—O17^iii^	111.4 (3)	O8—Si3—O7	111.97 (11)
O5—Li2—O17^iii^	133.6 (3)	O9—Si3—O7	108.15 (11)
O15^x^—Ti—O3^iii^	89.64 (8)	O10—Si3—O7	104.31 (11)
O15^x^—Ti—O8^ii^	90.65 (8)	O12^xi^—Si4—O11	113.88 (10)
O3^iii^—Ti—O8^ii^	90.97 (8)	O12^xi^—Si4—O10	109.69 (10)
O15^x^—Ti—O17^i^	85.19 (8)	O11—Si4—O10	111.83 (11)
O3^iii^—Ti—O17^i^	173.77 (8)	O12^xi^—Si4—O13	109.30 (11)
O8^ii^—Ti—O17^i^	92.55 (8)	O11—Si4—O13	109.83 (11)
O15^x^—Ti—O5	174.56 (8)	O10—Si4—O13	101.60 (10)
O3^iii^—Ti—O5	92.76 (8)	O15—Si5—O14	114.59 (11)
O8^ii^—Ti—O5	84.44 (8)	O15—Si5—O16	106.47 (11)
O17^i^—Ti—O5	92.69 (8)	O14—Si5—O16	109.69 (11)
O15^x^—Ti—O12	93.66 (8)	O15—Si5—O13	111.35 (11)
O3^iii^—Ti—O12	85.62 (8)	O14—Si5—O13	109.23 (11)
O8^ii^—Ti—O12	174.48 (8)	O16—Si5—O13	105.04 (11)
O17^i^—Ti—O12	91.24 (8)	O17—Si6—O18	113.54 (11)
O5—Ti—O12	91.39 (8)	O17—Si6—O16	109.83 (11)
O3—Si1—O2	113.90 (11)	O18—Si6—O16	110.86 (11)
O3—Si1—O1	110.90 (11)	O17—Si6—O1	108.67 (11)
O2—Si1—O1	110.09 (11)	O18—Si6—O1	110.52 (11)
O3—Si1—O4	106.12 (11)	O16—Si6—O1	102.88 (11)
O2—Si1—O4	111.69 (11)		

Symmetry codes: (i) *x*−1/2, *y*−1/2, *z*; (ii) −*x*+1/2, *y*−1/2, −*z*+1/2; (iii) −*x*+1, *y*, −*z*+1/2; (iv) −*x*+1, −*y*+1, −*z*+1; (v) −*x*+1/2, −*y*+1/2, −*z*+1; (vi) −*x*+1, −*y*, −*z*+1; (vii) *x*−1/2, *y*+1/2, *z*; (viii) −*x*+1/2, −*y*+3/2, −*z*+1; (ix) −*x*, −*y*, −*z*+1; (x) −*x*+1, *y*−1, −*z*+1/2; (xi) *x*, *y*+1, *z*; (xii) *x*+1/2, *y*+1/2, *z*; (xiii) *x*+1/2, *y*−1/2, *z*; (xiv) −*x*+1/2, *y*+1/2, −*z*+1/2; (xv) *x*, *y*−1, *z*; (xvi) −*x*+1, *y*+1, −*z*+1/2.
